# Lightning Strike Presenting as Fatal Lung Contusion: A Case Report

**DOI:** 10.7759/cureus.33125

**Published:** 2022-12-30

**Authors:** Sujal Patel, Pallavi Yelne, Shilpa A Gaidhane, Sunil Kumar, Sourya Acharya, Divit Shah, Mansi Patel, Yogesh Kakde

**Affiliations:** 1 Department of Medicine, Jawaharlal Nehru Medical College, Datta Meghe Institute of Medical Sciences, Wardha, IND; 2 Department of Medicine, Datta Meghe Institute of Medical Sciences, Wardha, IND; 3 School of Epidemiology and Public Health, Jawaharlal Nehru Medical College, Datta Meghe Institute of Medical Sciences, Wardha, IND; 4 Department of Medicine, Jawaharlal Nehru Medical College, Wardha, IND; 5 Internal Medicine, Jawaharlal Nehru Medical College, Wardha, IND

**Keywords:** respiratory failure, rhabdomyolysis, pulmonary contusion, vocal cord palsy, lightening injury

## Abstract

Electrical injuries to humans from a lightning strike are associated with significant rates of morbidity and fatality. High-voltage injuries including those caused by lightning strikes are pulmonary edema, pulmonary contusion, acute respiratory distress syndrome, and pulmonary hemorrhages. Patients who get injured experience secondary trauma in addition to direct and indirect injury. In this report, we present the case of a 62-year-old male patient with complaints of shortness of breath, vomiting, and hoarseness of voice. The patient’s treatment included airway protection, antibiotics, corticosteroids, and supportive care; however, the patient did not survive due to a severe lung contusion.

## Introduction

Lightning is a life-threatening natural disaster with an electrical energy content of 100-300 million volts and produces heat at a temperature of about 3000 °C. Prior studies estimate that there are 0.09 to 0.12 lightning strikes per 100,000 individuals worldwide [1]. Around 0.2-1.7 deaths per million people are reportedly caused by lightning each year globally [2]. In India, lightning strikes are thought to be responsible for over 2500 fatalities every year [3].

Many lightning-related fatalities cause sudden cardiac death by either ventricular fibrillation or asystole. Among the survivors, neurological manifestations like encephalopathy, intracranial hemorrhage, and neuropathy, cardiac dysrhythmias, other forms of injuries like burns, tympanic and ocular impairment are common. Solid organ damage can occasionally result from lightning's blasting action [4]. The spleen, liver, lungs, and bowels can be harmed by blunt trauma from shock waves, falling, or being struck with some object.

In this study, we describe the case of a 62-year-old man who presented to the emergency room with recurrent laryngeal nerve palsy and pulmonary contusion after being struck by lightning on his farm.

## Case presentation

After being struck by lightning, a 62-year-old man lost consciousness and was taken to the emergency room. he had been working on a farm with his family members when the incident occurred. He regained consciousness after half an hour in the ambulance. At the time of arrival at the hospital, he complained of severe pain all over the body, hoarseness of voice, coughing, and regurgitation. The patient also complained of shortness of breath at rest, which was sudden in onset. He denied any history of hemoptysis, tuberculosis, smoking, asthma, and past hospitalization with similar complaints.

On admission, he was irritable, opened his eyes when hearing any voice, and was able to move all four limbs, with a power grade of 5/5. His respiratory rate was 34 breaths per minute, his pulse was 120 beats per minute, and his blood pressure was 90/60 mmHg. Saturation was 88% on room air. Respiratory examination revealed bilateral crepitations in the infra-axillary, infra-scapular, and mammary regions. A cardiovascular examination revealed no abnormalities. Per abdomen examination was normal. His ear examination revealed a rupture of the tympanic membrane on the right side, but the left ear showed no abnormality.

**Figure 1:**
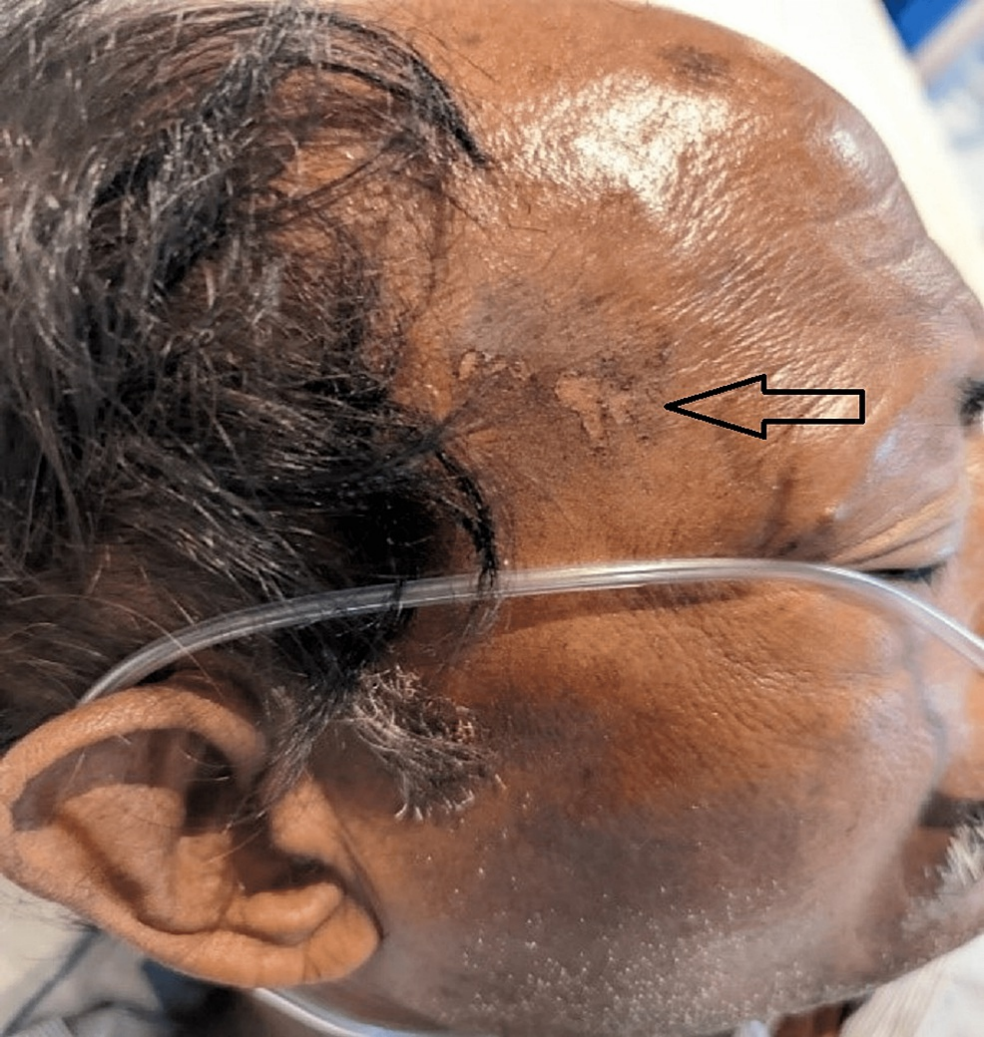
Entry wound of 2 x 2 cm in diameter on the right side of the forehead: a linear-shaped first-degree burn

 His physical examination revealed an entrance wound of 1 × 3 cm in diameter, on the right side of the forehead (Figure 1).

**Figure 2:**
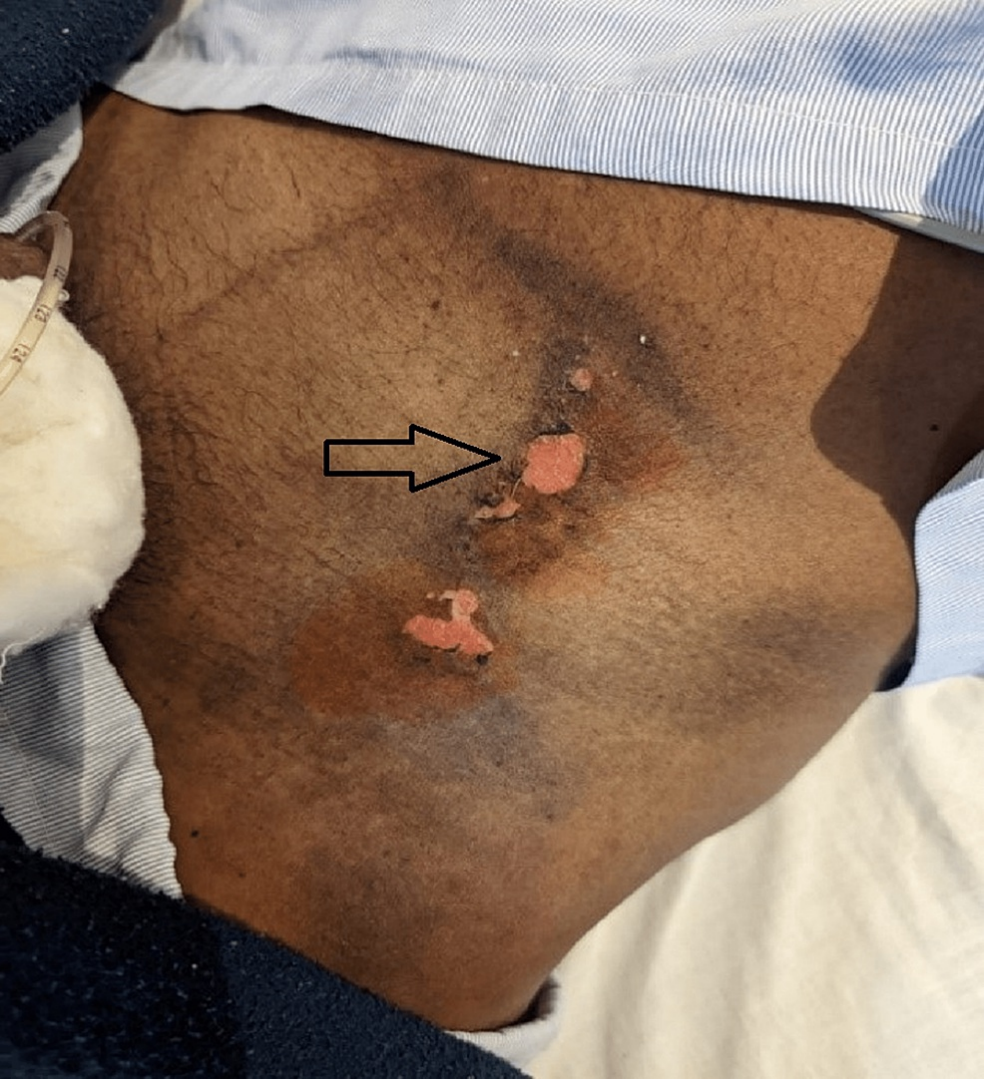
Exit wound of 2 x 3 cm in diameter, at the left iliac region: a linear-shaped first-degree burn showing ruptured bullae with a reddish base

The exit wound was 2 x 3 cm in diameter, at the left iliac region, a linear-shaped first-degree burn showing ruptured bullae with a reddish base (Figure 2). 

Video laryngoscopy was done in light of the hoarseness of voice and regurgitation, which revealed right vocal cord palsy with absent gag reflex (Video 1).

**Video 1:** Video laryngoscopy showing unilateral vocal cord palsy

All the routine investigations were done, and the findings are summarized in Table 1.

**Table 1:** Routine investigation findings in our patient

Investigation	Patient values	Reference values
Hemoglobin	10.4 g/dl	13-17 g/dl
White blood cells	23.3 × 10^3^/μl	4-10 × 10^3^/μl
Platelet count	1.08 × 10^4^/μl	1.5-4.10 × 10^4^/μl
Creatinine phosphokinase	1350 mg/dl	55-170 U/L
Serum creatinine	2.4 mg/dl	0.6-1.25 mg/dl
Serum urea	75 mg/dl	19-43 mg/dl
Serum sodium	152 meq/ltr	135-155 meq/L
Serum potassium	5.5 meq/ltr	3.5-5.1 meq/L
Random blood glucose	88 mg/dl	70-150 mg/dl
Total protein	7.4 gm/dl	6.3-8.2 gm/dl
Albumin	3 gm/dl	3.5-5.0 gm/dl
Aspartate aminotransferase	21 units/l	<50 units/l
Alanine aminotransferase	34 units/l	17-59 units/l
Alkaline phosphatase	120 IU/l	38-126 IU/l
Total bilirubin	2.3 mg/dl	0.2-1.3 mg/dl
Globulin	3.7 gm/dl	2.3-3.5 gm/dl

Creatinine kinase levels were monitored on a daily basis, as summarized in Table 2.

**Table 2:** Creatinine phosphokinase levels in our patient Daily creatinine phosphokinase levels; reference range: 0.6-1.25 mg/dl

Creatinine kinase levels	
Day 1	1350
Day 2	6395
Day 3	6003
Day 4	1547
Day 5	653
Day 6	1073
Day 7	635
Day 8	394
Day 9	193
Day 10	284
Day 11	178
Day 12	233
Day 13	101
Day 15	83
Day 17	44

**Figure 3:**
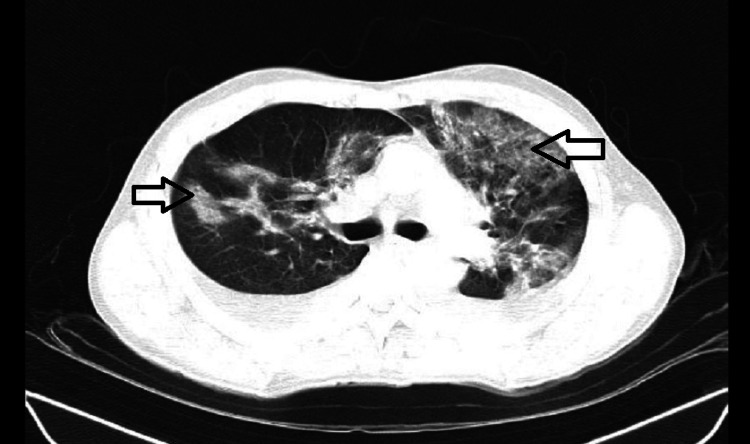
HRCT thorax revealing multifocal areas of ground glass appearance in both the upper lobe, left lingular lobe, and left lower lobe suggestive of possible pulmonary contusion HRCT: high-resolution computed tomography

**Figure 4:**
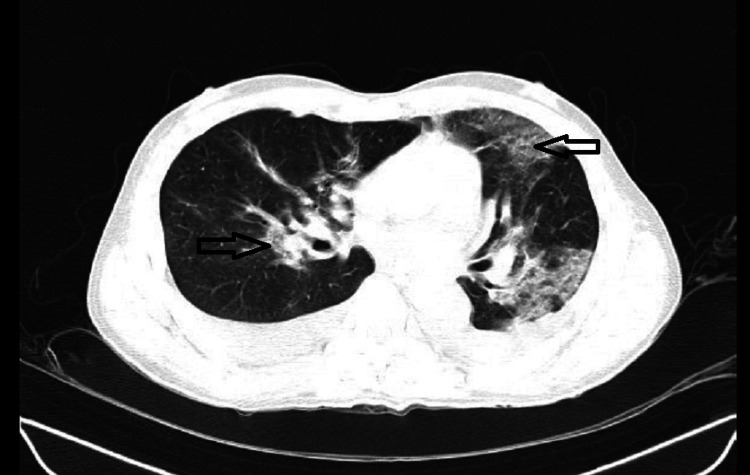
HRCT thorax revealing multifocal areas of ground glass appearance on both sides and pulmonary contusion on the left side HRCT: high-resolution computed tomography

Electrocardiography was suggestive of sinus tachycardia. Two-dimensional (2D) echo was suggestive of good biventricular function, left ventricle ejection fraction (LVEF) of 60% and all four chambers and valves were normal. A chest X-ray was done on admission, which revealed heterogeneous density increment in the left upper zone, left lower zone, and right upper zone. High-resolution chest CT (HRCT) thorax was done at the time of admission, which was suggestive of areas of ground glass opacities involving both upper lobes, left lower lobe, and left lingular lobe, indicating pulmonary parenchymal tear, and there were a few areas of pulmonary hemorrhage (Figures 3, 4).

Hydration with IV fluids and nasogastric tube feeding was started, and injectable tramadol and fentanyl were given for pain relief. Starting on hospital day one, the patient was kept on oxygen support for a period of seven days, after which the patient’s respiratory distress increased, and hence he was put on non-invasive ventilatory support. From day three of admission, the patient developed weakness in all four limbs and his power grade declined from 5/5 to 2/5 due to rhabdomyolysis-induced myositis. Electromyography was also done for confirmation, which was suggestive of a decreased duration of motor unit potentials.

For myopathy, steroid methylprednisolone 1 g was started and given for five days. After five days on steroids, his power improved to grade 4, and other supportive medications were started. During the second week, his oxygen saturation was maintained on non-invasive ventilation (NIV). However, during the third week of admission, arterial blood gas (ABG) monitoring revealed hypoxia with pO_2_ of 70%, pH of 7.202, CO_^2^_ of 44%, and base deficit of -10; hence, the patient was intubated in light of hypoxia, tachypnoea, and tachycardia and was put on a mechanical ventilator with volume control mode with FiO_2_ of 100%, peep of 6, vital capacity of 350, and RR of 20/min. Consecutive ABG monitoring was suggestive of pO_2_ of 95%, pH of 7.351, and CO_2_ of 40%.

In view of the long-standing mechanical ventilator support, a tracheostomy was planned on the seventh day of intubation. Cultures were sent of ET tube secretion; sputum culture and blood and urine cultures were suggestive of no growth after 48 hours of incubation. A concurrent chest radiogram suggested no new changes with regard to ventilator-acquired pneumonia. Despite aggressive medical management, the patient’s condition kept deteriorating and he eventually succumbed to respiratory failure on the 39th day of admission.

## Discussion

Lightening causes injury by way of five different mechanisms: thermal, electrical, explosive, magnetic field, and blunt damage from falls; however, the precise mechanism behind lightning injury remains unknown. The most serious clinical manifestation is most likely to result from direct hits [5]. The second mechanism produces a blast wave as a result of abrupt and significant changes in temperature. The blastic injury effect of a lightning strike can cause myocardial injury, lung contusion, lung parenchymal tear, major vascular rupture, intestine rupture, tympanic membrane rupture, and ocular damage [6]. In our case, lightning's electrical, thermal, and explosive effects all contributed to skin burns.

A pulmonary contusion is defined as a lung injury caused by a blunt explosive event without any accompanying chest wall injuries [7]. The lung tissue is not ripped or torn in pulmonary contusion cases, but blood and other fluids do pool there because of injury to the alveolar capillaries. Clinical indicators might vary significantly in terms of their severity and timing, which is unusual. Hypoxia and hypercarbia-related respiratory distress frequently manifests gradually and reaches their peak in around 72 hours [8,9]. When lightning hits, symptoms are often classified into immediate symptoms and symptoms that appear after a period of time, as in our case.

Moulson [8] described the first case of pulmonary contusion resulting from blastic injury in a lightning strike case without any burn injuries. According to Ohashi et al. [10], injuries such as cerebral hemorrhage, pulmonary hemorrhage, and solid organ rupture can be caused by falls or the lightning strike's current effects. The concussive action of rapidly expanding steam created by superheating water on the body surface of a wet person is what causes these injuries. Rapid surface flashover decreases internal energy dissipation and increases the likelihood of survival. We believe that in our case, the pulmonary contusion and hemorrhage were caused by the blastic effect of lightning that resulted from a quick surface flashover.

Tolunay et al. have reported the case of a 15-year-old male patient who was admitted to the emergency department due to a lightning strike in open terrain; even though the patient initially did not have any complaints, he developed breathlessness on the fourth day and was managed with oxygen therapy, and was discharged on 10th day with advice to follow up [11].

Uzel Şener et al. have described a case of a 19-year-old male patient who presented with a history of a lightning strike and developed cardiomyopathy with low ejection fraction; HRCT revealed bilateral pleural effusion and there were patchy consolidations in the upper lobes and areas of ground glass opacities around it, which were treated with oxygen therapy, steroids, antibiotics, and supportive measures. The patient was discharged with instructions to follow up after 10 days [12].

Schleich et al. have reported a case of a 23-year-old male patient who presented with severe third-degree burns on the chest who developed pulmonary embolism confirmed on CT angiography and was treated with heparin, antibiotics, and supportive care with ventilator support and systemic steroids. The patient recovered after a hospital stay of two months and was subsequently discharged [13]. The details of the studies mentioned are summarized in Table 3.

**Table 3:** Summary of previous studies on lung contusion due to lightning HRCT: high-resolution chest computed tomography

Age/sex	Presentation	X-ray chest finding	HRCT thorax finding	Ventilator Support	Outcome	Treatment	Reference
15 years/male	Multiple abrasions, breathing difficulty	No pathology	Ground-glass opacity and areas of consolidation	No support	Discharge for follow-up	Oxygen therapy was given	[11]
19 years/male	Severe abdominal pain and breathing difficulty	Not done	Bilateral pleural effusion; patchy consolidations in the upper lobes and areas of ground-glass opacities around it	No support needed	Discharge	Antibiotic support with systemic steroids and oxygen therapy	[12]
23 years/male	Severe third-degree burns on the upper aspect of the abdomen and chest	No pathology	Pulmonary embolism in the right lower lobe with bilateral pneumonia	Yes, on admission	Discharge	Fluid resuscitation and supportive treatment with management of pulmonary embolism	[13]

## Conclusions

Our case report presented a unique combination of complications caused by a lightning strike. Rhabdomyolysis is the most common complication, with laryngeal nerve palsy and lung contusion causing respiratory failure. Through a variety of causes, including blunt trauma, lightning strikes pose a serious hazard to human life. These patients must be closely monitored and should undergo a thorough respiratory evaluation on admission for early identification and management of any potential lung injury.
